# The complete mitochondrial genome of *Psychomantis borneensis* (Mantodea: Hymenopodidae)

**DOI:** 10.1080/23802359.2017.1419094

**Published:** 2017-12-21

**Authors:** Le-Ping Zhang, Yin-Yin Cai, Dan-Na Yu, Kenneth B. Storey, Jia-Yong Zhang

**Affiliations:** a College of Chemistry and Life Science, Zhejiang Normal University, Jinhua, Zhejiang Province, China;; b Key Lab of Wildlife Biotechnology, Conservation and Utilization of Zhejiang Province, Zhejiang Normal University, Jinhua, Zhejiang Province, China;; c Department of Biology, Carleton University, Ottawa, Canada

**Keywords:** Hymenopodidae, mitgenome, phylogeny, *Psychomantis borneensis*

## Abstract

The complete mitochondrial genome of *Psychomantis borneensis* (Mantodea: Hymenopodidae) was successfully sequenced. The mitochondrial genome is found to be 15,493 bp long and is a circular molecule containing 37 genes (13 protein-coding genes, 22 tRNAs, and 2 rRNAs), typically found in other mantis mitochondrial genomes. The AT content of the whole genome was 72.4% and the length of the control region was 697 bp with 79.9% AT content. A phylogenetic tree was constructed based on the BI and ML analysis of 16 species of Mantodea. The results showed that *P. borneensis* was a sister clade to (*Anaxarcha zhengi* +* Creobroter gemmata*) (Hymenopodidae). The monophyly of the family Mantidae and the genus *Theopompa*, *Hierodula*, and *Rhombodera* were not supported. The outcome of this study will provide a useful data for population genetics studies as well as serve as a tool for better characterizing phylogenetic analysis of Mantodea.

*Psychomantis borneens*is is currently assigned to the Acromantinae subfamily of Hymenopodidae. The phylogenetic relationships and classifications of Hymenopodidae species have not been studied extensively and up to date, only two Hymenopodidae species mitogenomes are published (Ye et al. 2016). Without extensive taxonomic sampling of DNA sequences, understanding the phylogenetic relationship between these species becomes virtually impossible (Svenson and Whiting 2009; Wieland 2013; Svenson et al. 2015). In this study, we sequenced the complete mitochondrial genome of *P. borneensis* (MG520077) to provide more molecular data that enables researchers to discuss the phylogenetic relationship of Hymenopodidae.

The sample of *P*. *borneensis* was collected from Borneo island, Indonesia in 2016, identified and stored at −40 °C in Zhang’s laboratory, College of Life Sciences and Chemistry, Zhejiang Normal University, China. Total DNA was extracted from leg muscle using DNeasy Blood and Tissue Kit (Qiagen, Germany). The universal primers used were the same as that used in the study by Simon et al. (2006) and Wang et al. (2016) with modifications. PCR products were sequenced in both directions by the primer-walking method by the Sangon Biotech Company (Shanghai, China).

The mitogenome of *P*. *borneensis* was found to be a circular 15,493 bp long molecule containing 37 genes (13 protein-coding genes, 22 tRNAs, and 2 rRNAs) and an A + T-rich region typically found in other insect mitogenomes (Cameron et al. 2006; Zhang et al. 2008; Ma et al. 2015; Ye et al. 2016; Tian et al. 2017; Zhang and Ye 2017). The average AT content of the whole genome was 72.4% and the length of the control region was 697 bp with 79.9% AT content. Most of the protein-coding genes used ATN (N represents A, T, C, G) as the initiation codon whereas the *COX1* and *ND5* genes were initiated with TTG and GTG, respectively. *COX2* and *ND5* used T–– as the termination codon while the remaining 11 protein-coding genes ended with TAA.

Phylogenetic relationships were constructed by following the Bayesian inference (BI) and maximum likelihood (ML) method using the MrBayes version 3.2 (Ronquist et al. 2012) and the RAxML version 8 programs, respectively (Stamatakis 2014). The mitochondrial genome of 19 different species including three cockroaches, *Blaptica dubia*, *Gromphadorhina portentosa*, and *Panchlota nivea* was used as the outgroups (Cheng et al. 2016).

The phylogenetic relationships inferred from the BI and ML analyses shared the similar topologies (Figure 1). *P. borneensis* was a sister clade to (*Anaxarcha zhengi* +* Creobroter gemmata*) (Hymenopodidae), hence, the monophyly of the family Hymenopodidae was supported in this study. Three Liturgusidae species clustered together and were sister to the remaining mantises while the topology ((*Humbertiella nada* +* Theopompa* sp.-YN) + *Theopompa* sp.-HN) indicated that *Theopompa* was a paraphyletic group. Mantidae was a polyphyletic assemblage which split into three clades. *Tenodera sinensis* (Mantidae) was a sister clade to the remaining Mantidae and Hymenopodidae species. *Statilia* sp. was a sister clade to *Mantis religiosa* and a total of six Paramantini species formed a clade. The monophyly of the genus *Hierodula* and *Rhombodera* were not supported.

**Figure 1.**
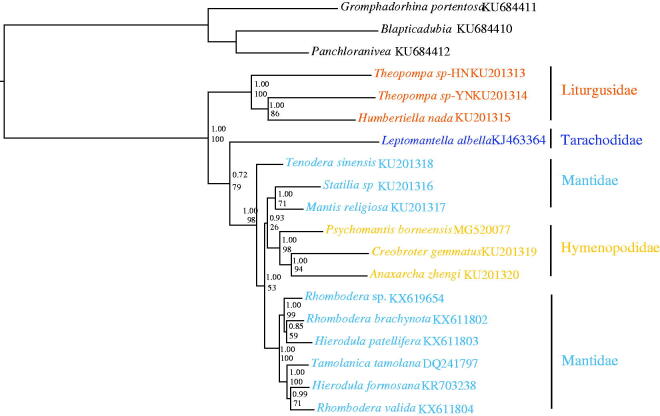
Phylogenetic tree of the relationships among 16 species of Mantodea (Cameron et al. 2006; Wang et al. 2016; Ye et al. 2016; Tian et al. 2017; Zhang and Ye 2017) based on the first and the second codon positions of the 13 mitochondrial protein-coding genes of 7112 nucleotides using *G. portentosa*, *B. dubia*, and *P. nivea* (Cheng et al. 2016) as outgroups. Numbers above branches specify posterior probabilities from Bayesian inference (BI) and bootstrap percentages from maximum likelihood (ML, 1000 replications) analyses. The GenBank numbers of all species are also shown.

## Nucleotide sequence accession number

The complete mitochondrial genome of *P*. *borneensis* has been assigned and deposited to GenBank with the following accession number MG520077.
